# Characterization and Genomic Analysis of *Pasteurella multocida* NQ01 Isolated from Yak in China

**DOI:** 10.3390/ani15233462

**Published:** 2025-12-01

**Authors:** Kewei Li, Haofang Yuan, Chao Jin, Muhammad Farhan Rahim, Xire Luosong, Tianwu An, Jiakui Li

**Affiliations:** 1 College of Animal Medicine, Huazhong Agricultural University, Wuhan 430070, China; lizangxi97@webmail.hzau.edu.cn (K.L.); yhf@webmail.hzau.edu.cn (H.Y.); 2024302010142@webmail.hzau.edu.cn (C.J.); farhan092@webmail.hzau.edu.cn (M.F.R.); 2 Xizang Veterinary Biological Pharmaceutical Factory, Lhasa 850003, China; m13908940656@163.com; 3 Sichuan Academy of Grassland Sciences, Chengdu 611731, China; 4 Jiangxia Laboratory, Wuhan 430200, China

**Keywords:** yak, hemorrhagic septicemia, *Pasteurella multocida*, whole-genome sequencing, virulence factor, intranasal infection, antimicrobial susceptibility

## Abstract

Hemorrhagic septicemia (HS) is a fulminant bovine disease across Asia and Africa, yet *Pasteurella multocida* (*P. multocida*) isolated from yak is poorly reported. We isolated strain NQ01 from a fatal HS case in Xizang, China and identified it as *P. multocida* B:2 by morphology, Gram stain, and PCR (*kmt1*^+^, *bcbD*^+^, LPS L2). NQO1 formed smooth, non-hemolytic colonies. After Gram staining, the cells appeared as red rods with bipolar staining. Antimicrobial testing showed broad susceptibility to β-lactams, aminoglycosides, tetracyclines, fluoroquinolones, midecamycin, florfenicol, polymyxin, and vancomycin, with resistance to metronidazole, trimethoprim sulfamethoxazole, and clindamycin. Streptomycin and ofloxacin had intermediate activity. In mice, the intraperitoneal and intranasal LD_50_ values were 40.64 CFU/mL and 9.53 × 10^6^ CFU/mL, respectively. The intranasal fatal cases were characterized by bacteremia with multifocal disseminated intravascular coagulation involving lung, liver, and spleen. The complete genome comprises a single 2.33 Mb chromosome (40.47% GC, 2115 CDS, no plasmids) with only one resistance gene (*Eco_EFTu_PLV*) and 28 virulence genes spanning adhesion (*tadA*, *rcpA*, *ppdD*, *pilB*, *tuf/tufA*, *htpB*, *PM_RS00430*, *PM_RS00425*, *PM_RS08640*), immune modulation (*lpxB/C/D*, *msbB*, *manB*, *rfaE/F*, *gmhA/lpcA*, *kdsA*, *pgi*, *wecA*, *galE*, *bexD’*, *ABZJ_RS06285*, *ABD1_RS00310*), and nutritional/metabolic factor (*hgbA*, *hemR*, *hemN*), plus a YadA-like factor. Phylogenetically, NQ01 clusters with regional B:2 bovine/yak isolates. Collectively, these data define NQ01 as a highly virulent, low-resistance yak isolate and a practical model for natural-route HS pathogenesis and targeted control in high-altitude pastoral settings yaks.

## Simple Summary

This study characterizes a highly virulent *Pasteurella multocida* strain (NQ01) linked to hemorrhagic septicemia (HS) in yaks from Xizang, China. NQ01 was typed as serotype B:2 and clustered phylogenetically with regional bovine and yak isolates, implying shared transmission across the plateau, where yak pasteurellosis remains a notable burden (individual prevalence 1.3%; herd-level > 15%). In BALB/c mice, NQ01 showed an unusually low intraperitoneal LD_50_ and retained lethality after intranasal exposure, underscoring robust pathogenicity via both systemic and respiratory routes. The strain was broadly susceptible to antimicrobials but resistant to metronidazole, trimethoprim–sulfamethoxazole, and clindamycin; only one resistance determinant (*Eco_EFTu_PLV*, conferring pulvomycin resistance) was detected, indicating a low overall AMR profile. Genome annotation revealed enrichment of functions supporting genetic stability and metal ion homeostasis, particularly iron, zinc, and magnesium, consistent with traits that promote colonization and virulence. Twenty-eight virulence factors were identified, including targets tied to protein synthesis (*tuf/tufA*) and cell-wall biogenesis (*pgi*). Genes implicated in capsule formation (*bexD′*, *galE* and polysaccharide loci) and LPS core/Lipid A assembly (*gmhA/lpcA*, *rfaE*, *rfaF*, *lpxB/C/D*, *msbB*, *manB*, *kdsA*, with contextual roles for *wecA*) highlight a strong surface-structure arsenal. Adhesion machinery included type IV pili/tad components (*ppdD*, *pilB*, *tadA*, *rcpA*) and a YadA-like factor, while iron acquisition systems (*hgbA*, *hemN*, *hemR*) indicate efficient heme utilization. Collectively, these data provide the first comprehensive genomic and phenotypic portrait of a yak HS-associated *P. multocida* from Xizang and point to concrete targets for control strategies.

## 1. Introduction

*Pasteurella multocida* (*P. multocida*) is an important bacterial pathogen of livestock and poultry, responsible for fowl cholera in birds, atrophic rhinitis in swine, and both bovine respiratory disease complex and hemorrhagic septicemia (HS) in cattle and buffalo. Historically, capsular typing by Carter grouped *P. multocida* into five serogroups (A, B, D, E, and F) [1]. Building on this, Heddleston’s scheme further differentiated strains into 16 serotypes (1–16) according to their lipopolysaccharide (LPS) antigens, providing a complementary framework for epidemiology and pathogenesis studies [2]. Notably, serogroups B and E are predominant causative agents of HS in cattle and buffalo; serogroups A and D are associated with pneumonia in cattle, sheep, and swine [3,4,5]. Additionally, serogroup A and F were reported as nasopharyngeal commensal pathogens linked to respiratory diseases in livestock, particularly inducing atrophic rhinitis in swine and rabbits [6,7]. Both capsular polysaccharides and LPS function as pivotal virulence factors: the capsule enhances bacterial invasiveness through anti-phagocytic properties, while LPS exacerbates tissue damage by activating host inflammatory cascades [8,9].

HS remains a major barrier to cattle production, owing to its peracute progression and high transmissibility. Outbreaks often sweep through herds so quickly that animals die in clusters before treatment can be applied [10]. In the acute form, death may occur within 24 h, typically preceded by a sudden high fever, labored breathing, and cyanosis of visible mucous membranes. The subacute form follows a slightly longer, 2–3-day course marked by prominent subcutaneous edema most notably in the mandibular and pectoral regions—alongside fever, anorexia, nasal discharge, hypersalivation, and dyspnea [11]. Globally, HS is responsible for substantial morbidity and mortality in cattle and buffalo populations, resulting in substantial economic losses [12,13,14]

The distribution and outbreak patterns of HS vary across different agro-climatic zones [15]. Yak is an endemic bovine species adapted to alpine regions, which predominantly inhabits the Qinghai–Xizang Plateau and peripheral high-altitude ecosystems [16]. Constrained by harsh environmental conditions and extensive husbandry practices, yak populations face formidable challenges in disease prevention and control [17]. Although a limited number of *P. multocida* genomes from yaks have been deposited in GenBank, detailed reports on the physiological and biochemical characteristics as well as the pathogenic mechanisms of these isolates remain scarce.

In 2020, the natural free-range yaks in Xizang showed clinical symptoms of cough and shortness of breath, high mortality appeared, and more than 100 yaks succumbed. A dominant strain was isolated from the lung of a dead yak, identified as *P. multocida* serotype B:L2. To further understand the potential pathogenic characteristics of this *P. multocida* strain, the whole-genome sequence was performed. Our study demonstrates that the isolate NQ01 exhibited high virulence, causing severe hemorrhagic septicemia in mice, which correlates with the carriage of multiple virulence genes. In contrast, this strain harbored only a single antimicrobial resistance gene and remained susceptible to the majority of antibiotics tested. Furthermore, whole-genome sequencing systematically elucidated the genetic blueprint of NQ01, thereby enriching the pathogenomics resources available for *P. multocida* research.

## 2. Materials and Methods

### 2.1. Bacteria Isolation and Culture Conditions

Fresh lung samples were aseptically collected from three moribund yaks, immediately frozen at −20 °C, and transported to our laboratory in Wuhan for pathogen isolation and culture. Several bacterial isolates exhibiting dominant growth were purified by streaking on tryptose soya agar (TSA) containing 5% defibrillated sheep blood, followed by incubation at 37 °C for 24 h. Subsequently, individual colonies were transferred to tryptose soya broth (TSB) containing 5% fetal bovine serum (FBS) at 37 °C for 24 h. Later, the strains were stored at −80 °C with 25% (*v*/*v*) glycerol. The morphological characteristics of NQ01 were analyzed using an optical microscope. Colonies were resuspended in Phosphate-Buffered Saline (PBS) buffer, dropped onto glass slides, and air-dried. Gram staining was subsequently performed (G1060, Solarbio, China), followed by microscopic examination.

### 2.2. Identification and Genotyping

Bacterial genomic DNA was extracted and purified from the bacterial cultures using a FastPure Bacteria DNA Isolation Mini Kit (DC103-01, Vazyme, Nanjing, China). To specifically detect *P. multocida* and serotypes, bacterial DNA was extracted for PCR analysis. The PCR reaction mixture contained a DNA template, 2 × Taq Master Mix (P113-03, Vazyme, China), sterile ultrapure water, and a set of primers synthesized by Tsingke Biotechnology Co., Ltd (Wuhan, China). The amplification primers and PCR reaction protocols are followed by the reported methods and shown in Table 1 and Table 2, respectively [18,19].

### 2.3. Antibiotic Susceptibility Testing

The stored NQ01 strain was incubated in TSB (with 5% FBS) at 37 °C for 24 h, then diluted to 1 × 10^8^ CFU/mL using sterile PBS. Antimicrobial susceptibility of the isolated strain was determined using the Kirby–Bauer disk diffusion method (Hangwei, Hangzhou China). Briefly, 100 µL of bacterial suspension was evenly spread onto TSA blood agar plates. Corresponding antibiotic disks including neomycin, doxycycline, cefalexin, amoxicillin, spectinomycin, enrofloxacin, florfenicol, streptomycin, gentamicin, ofloxacin, amikacin, trimethoprim-sulfamethoxazole, furazolidone, clindamycin, midecamycin, carbenicillin, ciprofloxacin, polymyxin, kanamycin, piperacillin, cefradine, vancomycin, cefoperazone, cefuroxime, ceftazidime, minocycline, and metronidazole were placed on the solid medium surface, followed by incubation at 37 °C for 24 h. The diameter of inhibition zones was measured with a caliper, and results were interpreted according to Clinical and Laboratory Standards Institute (CLSI) standards.

### 2.4. Median Lethal Dose Determination

Thirty-five 6-week-old BALB/c mice were randomly allocated to seven groups (*n* = 5 per group). A single *NQ01* colony was inoculated into 5 mL TSB and incubated at 37 °C for 12 h, after which the culture was serially diluted in sterile PBS. Lethal dose 50% (LD_50_) was determined by challenging mice via two routes, intraperitoneal (IP) injection and intranasal (IN) instillation, using graded inocula prepared from the dilution series. In the IN group, doses of 1.52 × 10^4^, 1.52 × 10^5^, and 1.52 × 10^6^ CFU per mouse were delivered in a volume of 20 µL. In the IP group, doses of 1.9, 3.8, and 5.7 CFU per mouse were administered in a volume of 0.1 mL. The control group received an IP injection of 0.1 mL of physiological saline. All animals were monitored for 7 days post inoculation. The LD_50_ was calculated using the Reed–Muench method. All experiments were conducted in accordance with the guidelines approved by the Laboratory Animal Centre of Huazhong Agricultural University (Approval number: 2025-0129).

### 2.5. Histopathological Analysis

The organisms from the experimental mice infected with a lethal dose were harvested following euthanasia, rinsed with PBS, and fixed in 4% paraformaldehyde for 48 h. Fixed tissues were embedded in paraffin, sectioned at 4 μm, and subjected to hematoxylin and eosin (H&E) staining after dehydration and dewaxing. The stained sections were examined under a light microscope.

### 2.6. Whole-Genome Sequencing and Genome Annotation of NQ01

The experimental procedure was performed following the standard protocol provided by Oxford Nanopore Technologies Co. Ltd. (Oxford, UK). The stored NQ01 strain was incubated in TSB (5% FBS) at 37 °C for 24 h. Bacterial cells were harvested by centrifugation at 4000 rpm for 2 min and washed three times with sterile PBS. Purified genomic DNA was obtained using SDS-based extraction method combined with purification columns. Genomic DNA quality was evaluated by agarose gel electrophoresis (1%), and purity and concentration were measured using a NanoDrop One spectrophotometer (A260/A280) and a Qubit 3.0 fluorometer. High-quality DNA was submitted to Wuhan Benagen Biotechnology Co., Ltd. (Wuhan, China) for whole-genome sequencing. Libraries were prepared and sequenced on both the Oxford Nanopore PromethION platform and the Illumina NovaSeq PE150 platform. Hybrid assemblies were generated with Unicycler (version 2.1.2) to produce a complete, high-accuracy bacterial genome. Coding-protein genes were predicted from the genome using Prokka software (version 1.1.2). The predicted gene sequences were subsequently subjected to BLAST (https://blast.ncbi.nlm.nih.gov/Blast.cgi) analysis against functional databases, including Clusters of Orthologous Groups (COG), Kyoto Encyclopedia of Genes and Genomes (KEGG), and the Gene Ontology (GO) for functional annotation. The Comprehensive Antibiotic Resistance Database (CARD) was utilized for the identification of potential antibiotic resistance genes with a minimum identity threshold of 90%. Virulence factors were screened against the full dataset of the Virulence Factors Database (VFDB) using thresholds of >70% identity and an E-value < 1 × 10^−5^.

### 2.7. Comparative Analysis of NQ01

Twenty-six complete genomes (with only one contig) of *P. multocida* strains isolated from bovines were retrieved from Genebank database. The phylogenetic analysis was constructed based on single nucleotide polymorphism (SNP) using Snippy v4.6.0; PM-1 was chosen as the reference genome. The strain FDAARGOS_218 was designated as the reference genome for *P. multocida*, and Pm70 is the first complete genome; both of them were also subjected to genomic alignment. A maximum likelihood phylogenetic tree was constructed from core SNPs using IQ-TREE v3.0.1 and subsequently visualized in Chiplot (https://www.chiplot.online/ accessed on 19 November 2025). The MLST genotypes were obtained at the website (http://pubmlst.org/pmultocida/ accessed on 19 November 2025). All genomes were annotated for structural and functional features using Prokka. Subsequently, the pan-genome was constructed with Panaroo in strict mode to compare the composition of core and accessory genes and to identify strain-specific gene clusters.

## 3. Result

### 3.1. Isolation and Identification of NQ01

**Figure 1:**
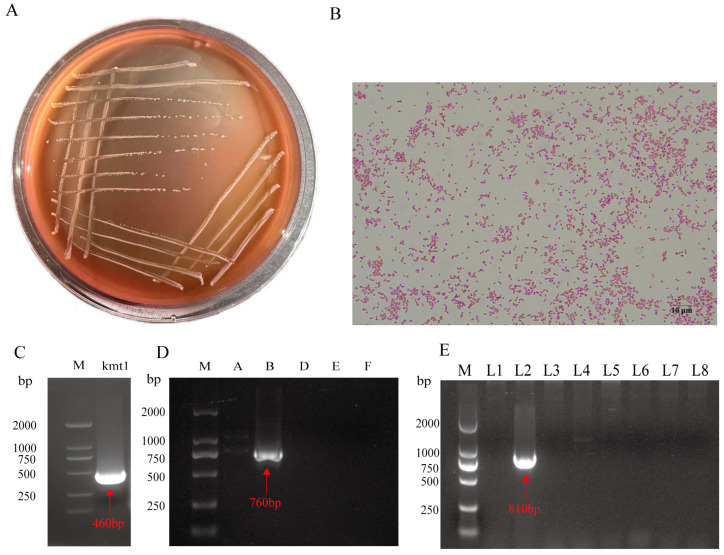
Culture characters and molecular types of *NQ01*. (**A**) *NQ01* cultured on TSA broth with 5% defibrillated sheep blood. (**B**) Microscopic examination at 1000x magnification following Gram staining. Results of PCR amplification targeting the species-specific gene *kmt1* (**C**), serogroup (**D**), and LPS genotype (**E**) in NQ01. M: DNA marker.

After 24 h of growth, the small greyish dominant colonies were selected from TSA agar containing 5% defibrillated sheep blood for identification. Following multiple rounds of subculturing and purification, smooth, moist, grayish-white translucent circular colonies were observed on the agar plates, with no discernible hemolytic activity (Figure 1A). Microscopic examination following Gram staining revealed rod-shaped cells displaying characteristic bipolar staining patterns and a pink coloration, consistent with Gram-negative bacteria (Figure 1B). Molecular characterization of the isolate was performed through PCR-based genotyping. The species-specific gene *kmt1* was successfully amplified as a 460 bp fragment (Figure 1C), confirming the isolate as *P. multocida*. Capsular typing revealed a 760 bp amplification product of the *bcbD* gene (Figure 1D), identifying the strain as serogroup B. Additionally, LPS genotyping demonstrated an 810 bp amplified fragment (Figure 1E), designating the LPS genotype as L2.

### 3.2. Antibiotic Susceptibility of NQ01

As shown in Table 3, antimicrobial susceptibility testing revealed that the *P. multocida* isolate exhibited sensitivity to most antibiotics tested. Notably, the isolate exhibited resistance to metronidazole, trimethoprim-sulfamethoxazole, and clindamycin.

### 3.3. Evaluated Pathogenicity of NQ01

The plateau phase of NQ01 was previously determined to be approximately 7.6 × 10^9^ CFU. By 36 h post-infection, several mice in the Pm group exhibited signs of lethargy, ruffled fur, and rapid breathing, in contrast to the normal behavior observed in the control group. By 7 days post-infection, the number of deaths in each group was recorded. Concurrently, all mice in the control group survived throughout the experimental period. Following intraperitoneal (IP) challenge, inocula of 5.7, 3.8, and 1.9 CFU produced mortality rates of 100% (5/5), 60% (3/5), and 0% (0/5), respectively, yielding an IP LD_50_ of 40.64 CFU/mL. In contrast, intranasal (IN) instillation with 1.52 × 10^6^, 1.52 × 10^5^, and 1.52 × 10^4^ CFU resulted in 100% (5/5), 40% (2/5), and 0% (0/5) mortality, corresponding to an IN LD_50_ of 9.53 × 10^6^ CFU/mL. Parameter estimates and goodness-of-fit metrics used in LD_50_ determination are summarized in Table 4.

### 3.4. Pathological Changes Induced by NQ01 Infection

**Figure 2:**
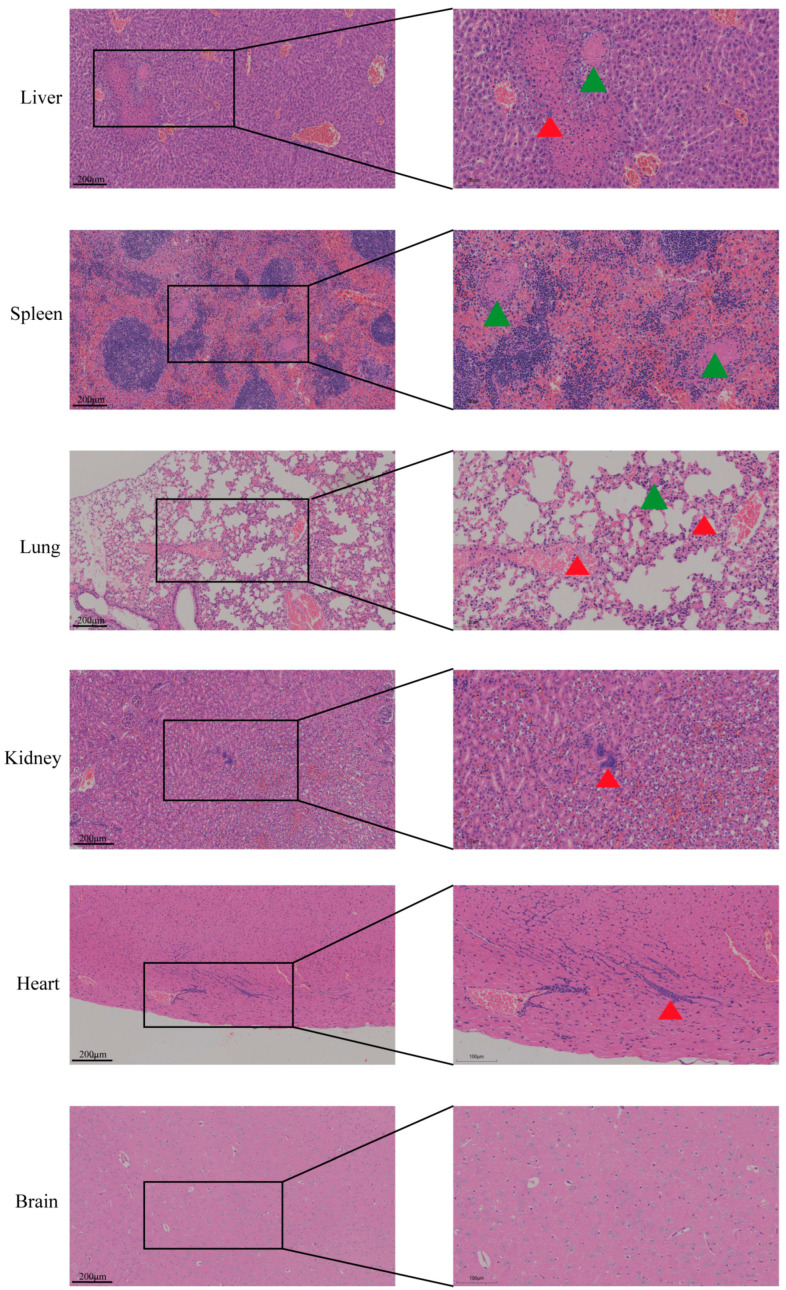
Pathological changes in mice. The mice infected with a lethal dose of *NQ01* were euthanized, and the major organs were assessed by H&E staining. The representative results are shown here; the red arrows indicate bacterial colonies, and green arrows indicate microthrombi.

As in Figure 2, NQ01 infection induced significant bacteremia-associated pathological changes in the major organs of mice. In the heart, sparse bacterial colonization was observed within the myocardial interstitium, accompanied by minor inflammatory cell infiltration adjacent to the coronary arteries. Scattered bacteria were also detected in the renal interstitium. The lungs, liver, and spleen exhibited multifocal disseminated intravascular coagulation (DIC). Pulmonary capillaries contained numerous microthrombi, while large veins showed bacterial colonies and inflammatory cell accumulation. In the liver, thrombus formation occurred in the central vein lumen, resulting in ischemic infarction of surrounding hepatocytes; bacterial colonies were present within the infarcted areas. The splenic red pulp demonstrated venous thrombus formation containing fibrinoid material mixed with leukocyte debris. No significant pathological changes were observed in brain tissue sections.

### 3.5. General Genomic Features of NQ01

**Figure 3:**
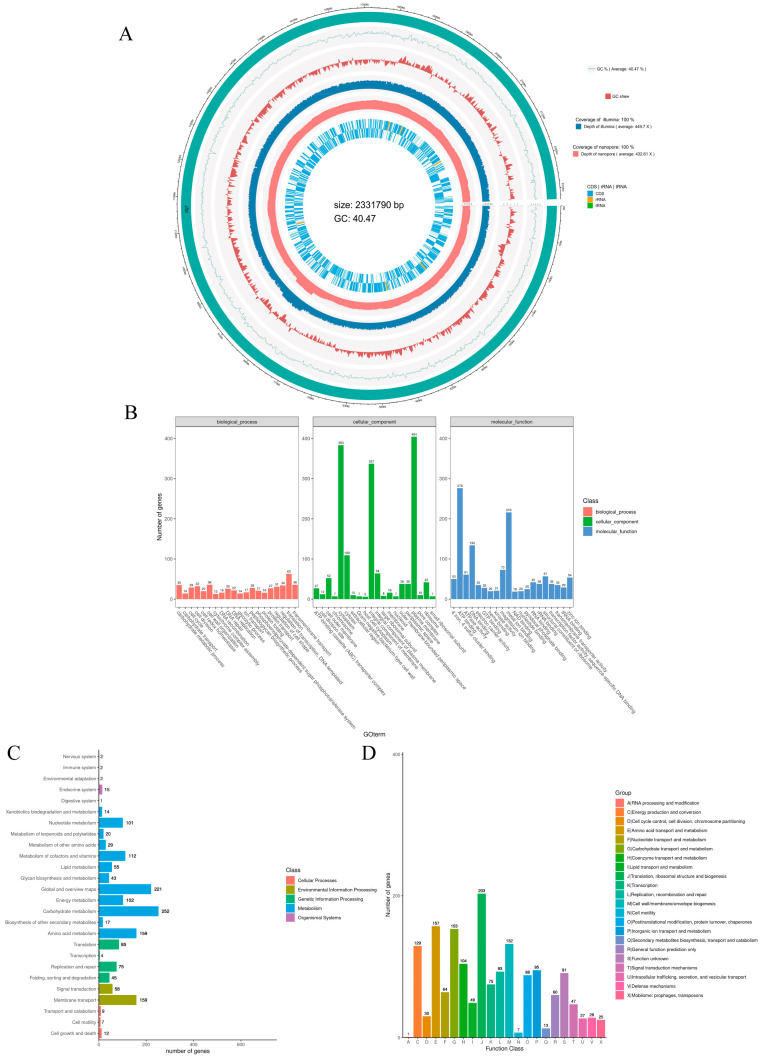
(**A**) Circular genome map of NQ01. From outer circle to inner, Circle 1: The genomic sequence information. Circle 2: GC content profile of the genome. Circle 3: GC skew profile of the genomic sequence. Circle 4: The second-generation sequencing (illumina) depth and coverage profile. Circle 5: The third-generation sequencing (nanopore) depth and coverage profile. Circle 6: Annotation of coding sequences (CDS) and non-coding RNA regions (rRNA, tRNA) in the reference genome. (**B**) GO annotation and classification of NQ01. The bar plot shows the number of genes assigned to GO terms within the three GO domains: biological process (salmon), cellular component (green), and molecular function (blue). The cellular component panel is dominated by intracellular terms, most notably cytoplasm (383 genes), cytosol (337), and ribosome (109), with additional contributions from plasma membrane (64) and membrane-associated compartments. In molecular function, the leading terms are structural constituent of ribosome (276 genes), metal-ion binding (216), and ATP binding (134), alongside several DNA/RNA-binding and transporter activities. Biological process terms are more dispersed; higher counts occur for transmembrane transport (63 genes) and multiple metabolism-, translation-, and cell-division-related processes (generally 20–40 genes each). The x-axis lists GO terms; the y-axis indicates gene counts. (**C**) KEGG pathway distribution of annotated genes. Horizontal bars show the number of genes assigned to each pathway; colors indicate the higher-level KEGG class (see legend). Metabolism dominates the annotation set, with the largest counts in carbohydrate metabolism (252 genes), global/overview maps (221), amino acid metabolism (159), membrane transport (159), energy metabolism (102), and nucleotide metabolism (101). Fewer genes fall under organismal systems and cellular processes (e.g., endocrine system, 15; cell growth and death, 12; cell motility, 7; immune and nervous systems, 2 each). The x-axis reports gene counts. (**D**) COG functional classification of annotated genes. Bars show the number of genes assigned to each COG category (A-X). The most represented groups are J: Translation, ribosomal structure and biogenesis (203 genes), E: Amino acid transport and metabolism (157), D: Cell cycle control, cell division, chromosome partitioning (153), N: Cell motility (132), and C: Energy production and conversion (129). Moderate counts include P: Posttranslational modification, protein turnover, chaperones (95), L: Replication, recombination and repair (93), S: Function unknown (91), and K: Transcription (75), whereas categories such as A: RNA processing and modification (1), Q: Secondary metabolite biosynthesis, transport and catabolism (13), and O: Cell wall/membrane/envelope biogenesis (7) are least represented. The y-axis indicates gene counts; category definitions are given in the legend.

The circular genome map of NQ01 illustrates genomic distribution features (Figure 3A). The complete genome of NQ01 consists of a single circular chromosome of 2,331,790 bp with a GC content of 40.47% and no plasmids. The chromosome encodes 2115 predicted protein-coding genes, 58 tRNA genes, and 19 rRNA genes.

The complete genome of NQ01 was annotated using the GO database, identifying a total of 1697 genes. These genes were further classified into three major categories: biological processes, cellular components, and molecular functions. The top 20 subcategories within each classification were visualized. In the biological process category, the annotated genes were predominantly associated with translation, transmembrane transport, cell wall organization, carbohydrate metabolic process, and regulation of transcription, DNA-template. For cellular components, 404 genes were annotated as encoding plasma membrane proteins, 383 for cytoplasm, 337 for integral component of membrane, 109 for cytosol, and 64 for integral component of plasma membrane. Additionally, genes encoding cell outer membrane, ribosome, and other related components were identified. In the molecular function category, 276 genes were associated with ATP binding, 216 with metal ion binding, and 134 with DNA binding. Other notable annotations included magnesium ion binding (73 genes), ATPase activity (61 genes), structural constituent of ribosome, zinc ion binding, and 4 iron 4 sulfur cluster binding (Figure 3B).

Annotation of the NQ01 genome using the KEGG database identified 1613 genes, which were categorized based on their involvement in various metabolic pathways (Figure 3C). The majority of annotated genes were associated with metabolism, followed by cellular processes, genetic information processing, environment information processing, and organismal systems. Within the metabolism category, 252 genes were linked to carbohydrate metabolism, 221 to global and overview maps, and 159 to amino acid metabolism. Other significant metabolic pathways included metabolisms of cofactors and vitamins, energy metabolism, nucleotide metabolism, and lipid metabolism. In the environment information processing category, 159 genes were associated with membrane transport and 58 with signal transduction. For genetic information processing, 85 genes were involved in translation, and 75 in replication and repair. In the organismal systems category, 12 genes were linked to cell growth and death, 9 to transport and catabolism, and 7 to cell motility. Fewer genes were associated with cellular processes, including 15 related to the endocrine system, 2 to the nervous and immune systems, and 1 to the digestive system.

The complete genome of NQ01 was annotated with 1619 genes using the COG database. As illustrated in Figure 3D, the most abundant functional category comprised 203 genes associated with translation, ribosomal structure, and biogenesis. This was followed by amino acid transport and metabolism, encompassing 157 genes. Additionally, 153 genes were annotated for carbohydrate transport and metabolism. The functional annotation further revealed that these genes are primarily involved in critical biological processes such as cell wall/membrane/envelope biogenesis and energy production and conversion. These findings collectively indicate that NQ01 possesses robust capabilities for material exchange and energy metabolism.

Notably, in comparison with the CARD database, we identified only one antimicrobial resistance gene, *Eco_EFTu_PLV*. This gene, identified as a sequence variant of *Escherichia coli* EF-Tu, belongs to the elfamycin-resistant EF-Tu family and confers resistance to Pulvomycin in the host bacterium. Furthermore, screening against the VFDB database revealed a total of 28 virulence genes (Table 5). Categorized by function, these included 9 genes associated with adhesion, 15 involved in immune modulation, and 3 related to nutrition and metabolism. Additionally, one virulence factor linked to the effector protein delivery system was identified.

### 3.6. Comparative Analysis Between P. multocida Isolates from Bovine

**Figure 4:**
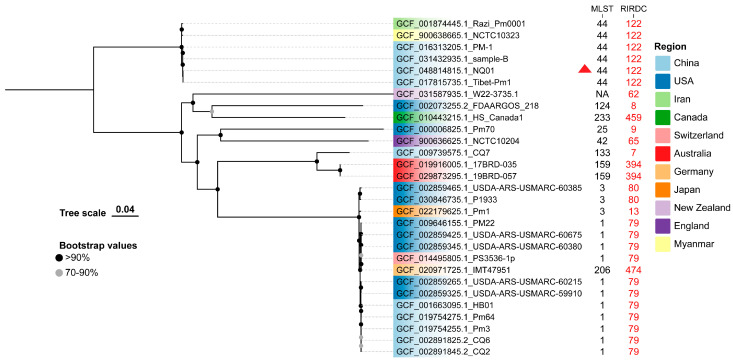
Core-genome SNP-based phylogenetic tree of NQ01 and other *P. multocida* isolates. The phylogenetic tree was generated by the ML method and based on SNP. The bootstraps value was 1000. The MLST and RIRDC MLST genotypes were listed behind the strain name, while different colors correspond different collected countries. Triangle indicates the isolated strain NQ01.

As illustrated in the phylogenetic tree, NQ01 clusters with Sample-B (China), Razi_Pm0001 (Iran), Tibet-Pm1 (China), PM-1 (China), and NCTC103023 (Myanmar), suggesting a close evolutionary relationship (Figure 4). Their MLST genotypes were ST44 (RIRDC ST122). This clade forms a monophyletic group and exhibits a more distant evolutionary relationship to other bovine-derived isolates (Figure 4). On the other hand, strains characterized as MLST ST1 (RIRDC ST79) clustered together and exhibited high SNP similarity to strains of MLST ST3 (RIRDC ST80 and ST83) and MLST ST206 (RIRDC ST474). In the pan-genome, 1834 core genes, 37 soft core genes, 787 shell genes, and 1295 cloud genes were identified. However, no strain-specific genes were detected.

## 4. Discussion

HS continues to cause significant losses in high-altitude pastoral systems, yet the role of *P. multocida* strains isolated from yaks remains underexplored. In this study, we isolated strain NQ01 from a yak in Xizang, China, and identified it as *P. multocida* serotype B:2, which has been frequently associated with severe HS outbreaks in bovine populations. Phylogenetic analysis placed NQ01 within a clade of regional *P. multocida* B: L2 strains from Asian countries, of which the MLST type were ST44 while the RIRDC MLST type were ST122. Notably, Tibet-Pm1 and PM-1 were also isolated from yaks in China, indicating a shared evolutionary origin and suggesting common transmission pathways across the plateau. Although the SNP-based phylogeny classified different genotypes into distinct clades, the pan-genome has a limited effective analysis on the accessory genes of NQ01, which seems have no genomic distinctiveness. With the isolates from yaks increasing, more genomic features will be further found. A recent study indicates that the prevalence of yak pasteurellosis was 1.30% at the individual level, and the group prevalence was over 15%, revealing that *P. multocida* is a direct threat to yak population health [20]. The identification of strain NQ01 further corroborates previous reports that *P. multocida* serotype B: L2 is the primary cause of HS in yaks, reinforcing the need for targeted control measures in these populations.

Intraperitoneal inoculation is the conventional approach for determining the LD_50_ of *P. multocida*. In our study, strain NQ01 displayed an unusually low IP LD_50_ in BALB/c mice, indicative of pronounced virulence. Notably, NQ01 also produced fatal disease after IN instillation, a route that better reflects upper respiratory exposure in natural infection. This is consistent with prior observations that some *P. multocida* strains are lethal by IP yet attenuated by the IN route [21,22]. Though the dissemination kinetics of NQ01 from the respiratory tract to the blood is unknown, these findings demonstrate that NQ01 maintains high pathogenicity across both exposure pathways and highlight its value as a model for research on HS, which serves as a significant basis for testing interventions that interrupt early colonization and systemic dissemination.

The antibiotic susceptibility testing revealed that NQ01 was susceptible to the majority of antibiotics but exhibited resistance to metronidazole, trimethoprim-sulfamethoxazole, and clindamycin. The antimicrobial spectrum of clindamycin was just limited to Gram-positive bacteria, and the absence of antibacterial activity was predictable. Similarly, metronidazole targets anaerobic bacteria, which explains its lack of antimicrobial activity against NQ01. The observed resistance to trimethoprim-sulfamethoxazole was unexpected, because only one antimicrobial resistance gene, called *Eco_EFTu_PLV*, was identified. This suggests the presence of alternative resistance mechanisms in NQ01. *Eco_EFTu_PLV* represents a sequence variant of *Escherichia coli* elongation (EF-Tu) and belongs to the elfamycin-resistant EF-Tu family, conferring resistance specifically to pulvomycin. Pulvomycin can inhibit the protein synthesis of EF-Tu. As the permeability of membrane enhanced, EF-Tu mutants may increase resistance to pulvomycin as an adaptive response to environmental stress [23]. Since pulvomycin is rarely employed in veterinary practice and was unavailable for testing, NQ01 demonstrates an overall low antimicrobial resistance profile. However, caution is warranted in the clinical application of antibiotics to mitigate the development of resistance.

The high conservation of the core genome in NQ01 suggests that it carries essential genes responsible for colonization and pathogenesis. Functional annotation of the NQ01 genome revealed key biological traits essential for its survival and virulence. A significant number of genes involved in genetic information processing were identified, supporting the stability of the genome and enhancing the bacterium’s ability to proliferate and adapt to environmental stresses. In NQ01, over 16% genes (276 genes) were annotated in the ATP binding category, which ensures bacterial homeostasis under hypoxic conditions, facilitating the adaptation to the plateau environment. Furthermore, the annotation across multiple databases revealed a substantial number of genes involved in substance metabolism and nutrient regulation, suggesting a robust nutrient acquisition capacity, which facilitated resource scavenging from the host and environment to support its growth and reproduction.

Previous research has shown that metal ions play a crucial role in bacterial colonization, growth, adhesion, and virulence, highlighting their importance in *P. multocida* pathogenesis [24,25]. Genes linked to metal ion binding were prominently represented, suggesting that NQ01 may have a strong capacity to compete for essential metal ions within the host. The ability to acquire and utilize iron is widely recognized as a key determinant of host virulence, as it supports bacterial growth, immune evasion, and successful infection establishment [26]. It has been confirmed that under iron-restricted conditions, the growth and virulence of *P. multocida* are attenuated, whereas low iron concentrations promote LPS synthesis, thereby enhancing its adhesive capability [27,28]. Consistent with previous studies, several virulence factors like *hgbA*, *hemN*, and *hemR* were also found in NQ01. While the *hgbA* gene enables the bacterium to bind hemoglobin and facilitates iron acquisition from the host, *hemN* and *hemR* are crucial for regulating heme biosynthesis, yet they function through different mechanisms [29,30]. HemN acts as a coproporphyrinogen III oxidase (CPO), catalyzing the conversion of coproporphyrinogen III to protoporphyrinogen IX, a key step in the heme biosynthesis pathway [31]. In contrast, HemR serves as a heme receptor, homologous to HgbA, and functions as a TonB-dependent receptor that facilitates the uptake of hemoglobin, playing a key role in the bacterium’s ability to utilize host-derived hemoglobin for iron acquisition [32]. These iron-regulatory mechanisms suggest that NQ01 has a highly efficient capacity for heme utilization and remarkable adaptability to the host environment. During the latent phase, HemN helps maintain essential ion metabolism, while, in response to tissue damage or hemorrhage, HemR and HgbA enable the rapid acquisition of iron, fueling the bacterium’s accelerated growth and contributing to the rapid onset and short disease course of HS caused by NQ01. This sophisticated iron regulation system ensures efficient oxygen transport and utilization, thereby enabling NQ01 to adapt to hypoxic conditions, both in the high-altitude environment and within the host. Additionally, genes encoding zinc-binding proteins were notably enriched in NQ01. Prior work indicates that zinc acquisition in *P. multocida* is coordinated by the iron-responsive regulator Fur and is essential for full virulence [33]. Although the roles of magnesium-binding proteins in this species are less well defined, the observed enrichment of Mg^2+^-binding functions in NQ01 suggests that magnesium homeostasis may likewise contribute to bacterial growth and pathogenic potential.

The virulence of *P. multocida* is multifactorial. In addition to iron-regulated and iron acquisition proteins, capsule, LPS, and fimbriae and other adhesins also contribute to the pathogenicity of *P. multocida.* While the exact roles of some of the identified virulence factors in NQ01 remain to be fully understood, the genomic analysis offers important insights into the bacterium’s biological functions. For instance, protein synthesis capacity and cell membrane integrity are essential prerequisites for bacterial physiological activities. *tuf* and *tufA*, which encode EF-Tu, are integral to protein synthesis. The concentration of EF-Tu within the cell is closely linked to both bacterial growth rate and translation efficiency, making it a critical factor for the bacterium’s proliferation [34]. The loss of *pgi* disrupts the synthesis of essential peptidoglycan precursors, leading to defects in the integrity of the bacterial cell envelope [35].

The capsule is a crucial determinant of *P. multocida* virulence, playing a central role in both serological classification and pathogenicity. Within the biosynthetic and export pathways for capsular formation, *bexD*’ is a core element of the bex export operon and mediates translocation of capsular polysaccharides across the bacterial envelope, while *galE* improves the formation of surface polysaccharides and their ability to adhere, ultimately leading to a significant increase in virulence [36,37]. In VFDB, *ABZJ_RS06285* and *ABD1_RS00310* are annotated as polysaccharide biosynthesis proteins (homologs characterized in *Acinetobacter baumannii*), implicating them as putative nodes in capsular assembly; however, further validation in NQO1 is still required. Together, these loci in NQ01 argue for a strong genetic capacity for capsular production and thus heightened virulence.

In parallel, LPS remains a central antigenic and structural determinant in Gram-negative pathogens, including *P. multocida*. Within the inner-core pathway, *gmhA/lpcA* encodes a phosphoheptose isomerase that supplies ADP-L-glycero-D-manno-heptose precursors, while *rfaE* (ADP-heptose synthase) and *rfaF* (ADP-heptose-LPS heptosyltransferase II) sequentially catalyze heptose synthesis and transfer to the core oligosaccharide. The coordinated activity of these enzymes is therefore pivotal for proper LPS core assembly and, by extension, outer-membrane integrity and immune interaction [38,39]. The catalytic enzymes encoded by *lpxB*, *lpxC*, *lpxD*, *msbB*, and *manB* are involved in regulating Lipid A synthesis [40,41,42,43]. *kdsA* encodes KDO 8-phosphate synthase, which catalyzes the first committed step in 3-deoxy-D-manno-oct-2-ulosonic acid (KDO) biosynthesis. Because KDO is essential for assembling the Lipid A core region of LPS, *kdsA* represents a key regulatory node in outer-membrane biogenesis and an attractive antibacterial drug target [44]. As reported in *Actinobacillus pleuropneumoniae*, *wecA* has been demonstrated to modulate O-antigen synthesis, thereby influencing bacterial growth and stress resistance; its deletion attenuates bacterial virulence [45]. Although LPS in *P. multocida* lacks an O-antigen component, a study in *Glaesserella parasuis* (another member of the Pasteurellaceae family) revealed significant upregulation of *wecA* in a ΔqseC mutant, suggesting its potential role in maintaining biofilm homeostasis [46].

Fimbriae and other adhesins are widely recognized as virulence factors that directly influence bacterial adhesion, motility, and macromolecule uptake [47,48]. In strain NQ01, several genes associated with fimbrial systems were identified. Previous research has demonstrated that PpdD is an essential structural component of Type IV pili and that mutants lacking *ppdD* show a marked reduction in cellular adhesion. This highlights the critical role of PpdD in promoting bacterial attachment and contributing to virulence [49,50]. *pilB* encodes an extension ATPase that is vital for the polymerization of Type IV pilin monomers, driving the elongation of pili. This process is crucial for the proper assembly and function of Type IV pili, which are essential for bacterial motility, adhesion, and virulence [51,52]. *PM_RS00425* is also annotated in the VFDB as having a function similar to *pilB*. Both *tadA* and *rcpA* are essential components of the tad locus. While the RcpA protein, encoded by *rcpA*, assembles the tad pili, the CpaF protein, encoded by *tadA*, drives their cyclic extension and retraction, which is important for pili function and bacterial adherence [53,54,55]. *PM_RS00430* encodes a protein from the Type II secretion system F family, which is functionally linked to the activities of Type IV pili. Additionally, the virulence factor *PM_RS08160*, which encodes a YadA-like protein, has been shown to play a crucial role in host cell adhesion and invasion, further contributing to the pathogenicity of *P. multocida* [56].

To the best of our knowledge, this study provides the first comprehensive characterization of a *P. multocida* strain associated with HS in yaks from Xizang, China. Through detailed clinical and genomic analysis of NQ01, we have uncovered key insights into the mechanisms driving HS pathogenesis. These findings lay the groundwork for developing targeted control strategies for HS in the region and enhance our understanding of *P. multocida* infection dynamics in yak populations.

## 5. Conclusions

This study offers the first in-depth characterization of a *P. multocida* strain associated with HS in yaks from Xizang, China. The isolation and detailed analysis of strain NQ01 highlight the significant pathogenic potential of *P. multocida* serotype B: L2, a major cause of disease in yak populations. Our findings emphasize the role of genomic features, such as capsular synthesis, LPS structure, and metal ion acquisition systems, in driving the virulence of NQ01. The identification of key virulence factors, including type IV pili and heme acquisition mechanisms, provides valuable insights into the molecular underpinnings of HS pathogenesis. Additionally, the low LD_50_ observed in both intraperitoneal and intranasal infection models demonstrates the relevance of NQ01 for studying natural infection routes. The antimicrobial susceptibility profile of NQ01 suggests that targeted therapeutic strategies could be developed to manage HS in high-altitude pastoral systems, while judicious antibiotic stewardship is crucial to prevent the emergence of resistance. Overall, this study lays a solid foundation for further research on the epidemiology and control of *P. multocida* in yaks, contributing to the development of more effective, region-specific measures for controlling HS in livestock.

## Figures and Tables

**Table 1:** Primers used in LPS and capsular genotyping of *Pasteurella multocida*.

Description Tested Features	Gene	Primers	Sequences (5′-3′)	Product Size (bp)
*P. multocida*	*Kmt1*	F-kmt	ATCCGCTATTATCCAGTGG	460
		R-kmt	GCTGTAAACGAACTCGCCA	
Serogroup A	*hyaD-hyaC*	F-A	TGCCAAATCGCAGTCAG	1044
		R-A	TTGCCATCATTGTCAGTG	
Serogroup B	*bcbD*	F-B	CATTCTATCCAAGCTCCACC	760
		R-B	GCCCGAGAGTTTCAATCC	
Serogroup D	*dcbF*	F-D	TTACACTAAAGCTCCAGGAGCCC	657
		R-D	CATCCACCACTCAACCATATCAG	
Serogroup E	*ecbJ*	F-E	TCCGCAGAAATTATTGACTC	511
		R-E	GCTTGCTGCTTGATTTTGTC	
Serogroup F	*fcbD*	F-F	AATCGGAAACGCAGAAATCAG	851
		R-F	TTCCGCCGTCAATTACTCG	
LPS type 1	*pcgD-* *pcgB*	F-1	ACATTCGAGATAATACACCCG	1307
		R-1	ATTGGAGCACCCTAGTAACCC	
LPS type 2	*nctA*	F-2	CTTAAAGTAACACTCGCTATTGC	810
		R-2	TTTGATTTCCCTTGGGATAGC	
LPS type 3	*gatF*	F-3	TGCAGGCAGAGAGTTGATAAACCATC	474
		R-3	CAAAGATTGGTTCAAATCTGAATGGA	
LPS type 4	*latB*	F-4	TTTCCATAGATTACCAATGCCG	550
		R-4	CTTCTAGTGGTAGTCTAATGTCGACC	
LPS type 5	*rmlA* *rmlC*	F-5	AGATTGCATGGCAAATGGC	1175
		R-5	CAATCCTCGTAAGACCCCC	
LPS type 6	*nctB*	F-6	TCTTTATAATTATACTCTCCCAAGG	668
		R-6	AATGAAGGTAAAAAGAGATAGCTGGAG	
LPS type 7	*ppgB*	F-7	CCTAATTTATATCTCTCCCC	931
		R-7	CTAATATATAACCCACCAACGC	
LPS type 8	*natG*	F-8	GAGAGTTACAAAAATGATCGGC	225
		R-8	TCCTGGTTCATATAGGTAGG	

**Table 2:** PCR amplification system for isolated bacterial DNA.

Temperature (°C)	Time
94	5 min
94	30 s for 35 cycles
55	30 s for 35 cycles
72	2 min for 35 cycles
72	10 min

**Table 3:** Antibiogram assay of NQ01.

Category	Antibiotics	Concentration (per Piece)	Susceptibility
β-lactam	cefalexin	30 μg	S
	cefradine	30 μg	S
	cefoperazone	75 μg	S
	cefuroxime	30 μg	S
	ceftazidime	30 μg	S
	amoxicillin	20 μg	S
	carbenicillin	100 μg	S
	piperacillin	100 μg	S
aminoglycoside	neomycin	30 μg	S
	kanamycin	30 μg	S
	gentamicin	10 μg	S
	spectinomycin	100 μg	S
	amikacin	30 μg	S
	streptomycin	10 μg	I
tetracycline	doxycycline	30 μg	S
	minocycline	30 μg	S
quinolone	enrofloxacin	10 μg	S
	ciprofloxacin	5 μg	S
	ofloxacin	5 μg	I
macrolide	medemycin	30 μg	S
chloramphenicol	florfenicol	30 μg	S
polypeptide	polymyxin	300 IU	S
	vancomycin	30 μg	S
nitroimidazole	metronidazole	5 μg	R
sulfonamide	trimethoprim-sulfamethoxazole	25 μg	R
lincosamide	clindamycin	2 μg	R

**Table 4:** Determination of LD_50_ for Pasteurella multocida NQ01 in BALB/c mice via intraperitoneal (IP) and intranasal (IN) routes.

Route of Administration	Dose (CFU/Mouse)	Survival	Survival Rate (%)
Control	0	5/5	100%
Intraperitoneal injection	1.9	5/5	100%
	3.8	2/5	40%
	5.7	0/5	0
Intranasal instillation	1.52 × 10^4^	5/5	100%
	1.52 × 10^5^	3/5	60%
	1.52 × 10^6^	0/5	0

**Table 5:** Virulence genes in NQ01.

Category	Gene	Function	Terms
Adherence	*htpB*	Hsp60 heat shock protein	3
	*tadA*	TadA pilin	1
	*rcpA*	RcpA pilus assembly proteins	1
	*PM_RS00430*	type II secretion system F family protein	1
	*PM_RS00425*	GspE/PulE family protein	1
	*pilB*	GspE/PulE family protein	1
	*ppdD*	prepilin peptidase-dependent pilin	1
	*tufA/tuf*	elongation factor Tu	6
	*PM_RS08640*	ComEA family DNA-binding protein	1
Immune modulation	*manB/yhxB*	Phosphomannomutase	4
	*ABZJ_RS06285*	Capsular polysaccharides synthesize proteins	1
	*ABD1_RS00310*	Capsular polysaccharides synthesize proteins	1
	*bexD’*	Capsular polysaccharides synthesize proteins	1
	*msbB*	Lipid A biosynthesis acyltransferase	4
	*pgi*	Glucose-6-phosphate isomerase	2
	*wecA*	Undecaprenyl-phosphate alpha-N-acetylglucosaminyl 1-phosphate transferase	1
	*kdsA*	2-dehydro-3-deoxyphosphooctonate aldolase	6
	*rfaE*	ADP-heptose synthetase	2
	*galE*	Udp-glucose 4-epimerase	2
	*lpxC*	UDP-3-O-acyl-N-acetylglucosamine deacetylase	4
	*gmhA/lpcA*	Phosphoheptose isomerase	3
	*lpxB*	Lipid-A-disaccharide synthase	2
	*lpxD*	UDP-3-O-(3-hydroxymyristoyl)glucosamine N-acyltransferase	2
	*rfaF*	ADP-heptose--LPS heptosyltransferase II	4
Nutritional/metabolic factor	*hgbA*	Hemoglobin-binding protein A	1
	*hemR*	Hemin receptor	2
	*hemN*	Oxygen-independent coproporphyrinogen III oxidase	4
Effector delivery system	*PM_RS08160*	YadA-like Protein	2

## Data Availability

The raw data supporting the conclusions of this article will be made available by the authors on request.

## References

[1] Carter G.R. (1955). Studies on Pasteurella multocida. I. A hemagglutination test for the identification of serological types. Am. J. Vet. Res..

[2] Heddleston K.L., Gallagher J.E., Rebers P.A. (1972). Fowl cholera: Gel diffusion precipitin test for serotyping Pasteruella multocida from avian species. Avian Dis..

[3] Oh Y.H., Moon D.C., Lee Y.J., Hyun B.H., Lim S.K. (2019). Genetic and phenotypic characterization of tetracycline-resistant Pasteurella multocida isolated from pigs. Vet. Microbiol..

[4] Fegan J.E., Waeckerlin R.C., Tesfaw L., Islam E.A., Deresse G., Dufera D., Assefa E., Woldemedhin W., Legesse A., Akalu M. (2024). Developing a PmSLP3-based vaccine formulation that provides robust long-lasting protection against hemorrhagic septicemia-causing serogroup B and E strains of Pasteurella multocida in cattle. Front. Immunol..

[5] Petruzzi B., Briggs R.E., Tatum F.M., Swords W.E., De Castro C., Molinaro A., Inzana T.J. (2017). Capsular Polysaccharide Interferes with Biofilm Formation by Pasteurella multocida Serogroup A. mBio.

[6] Liu H., Zhao Z., Xi X., Xue Q., Long T., Xue Y. (2017). Occurrence of Pasteurella multocida among pigs with respiratory disease in China between 2011 and 2015. Ir. Vet. J..

[7] Wang J., Sun S., Chen Y., Chen D., Sang L., Xie X. (2022). Pathogenic and genomic characterisation of a rabbit sourced Pasteurella multocida serogroup F isolate s4. BMC Vet. Res..

[8] Hildebrand D., Heeg K., Kubatzky K.F. (2011). Pasteurella multocida toxin-stimulated osteoclast differentiation is B cell dependent. Infect. Immun..

[9] Chakraborty S., Kloos B., Roetz N., Schmidt S., Eigenbrod T., Kamitani S., Kubatzky K.F. (2018). Influence of Pasteurella multocida Toxin on the differentiation of dendritic cells into osteoclasts. Immunobiology.

[10] Almoheer R., Abd Wahid M.E., Zakaria H.A., Jonet M.A.B., Al-Shaibani M.M., Al-Gheethi A., Addis S.N.K. (2022). Spatial, Temporal, and Demographic Patterns in the Prevalence of Hemorrhagic Septicemia in 41 Countries in 2005–2019: A Systematic Analysis with Special Focus on the Potential Development of a New-Generation Vaccine. Vaccines.

[11] Lestari T.D., Khairullah A.R., Damayanti R., Mulyati S., Rimayanti R., Hernawati T., Utama S., Kusuma Wardhani B.W., Wibowo S., Ariani Kurniasih D.A. (2025). Hemorrhagic septicemia: A major threat to livestock health. Open Vet. J..

[12] Govindaraj G., Krishnamoorthy P., Nethrayini K.R., Shalini R., Rahman H. (2017). Epidemiological features and financial loss due to clinically diagnosed Haemorrhagic Septicemia in bovines in Karnataka, India. Prev. Vet. Med..

[13] Mahboob S., Ullah N., Farhan Ul Haque M., Rauf W., Iqbal M., Ali A., Rahman M. (2023). Genomic characterization and comparative genomic analysis of HS-associated Pasteurella multocida serotype B:2 strains from Pakistan. BMC Genom..

[14] Maddock K.J., Stenger B.L.S., Pecoraro H.L., Roberts J.C., Loy J.D., Webb B.T. (2025). Hemorrhagic septicemia in the United States: Molecular characterization of isolates and comparison to a global collection. J. Vet. Diagn. Investig..

[15] Chanda M.M., Purse B.V., Hemadri D., Patil S.S., Yogisharadhya R., Prajapati A., Shivachandra S.B. (2024). Spatial and temporal analysis of haemorrhagic septicaemia outbreaks in India over three decades (1987–2016). Sci. Rep..

[16] Mo Q., Nawaz S., Kulyar M.F., Li K., Li Y., Zhang Z., Rahim M.F., Ahmed A.E., Ijaz F., Li J. (2024). Exploring the intricacies of Pasteurella multocida dynamics in high-altitude livestock and its consequences for bovine health: A personal exploration of the yak paradox. Microb. Pathog..

[17] Qiu Q., Zhang G., Ma T., Qian W., Wang J., Ye Z., Cao C., Hu Q., Kim J., Larkin D.M. (2012). The yak genome and adaptation to life at high altitude. Nat. Genet..

[18] Townsend K.M., Boyce J.D., Chung J.Y., Frost A.J., Adler B. (2001). Genetic organization of Pasteurella multocida cap Loci and development of a multiplex capsular PCR typing system. J. Clin. Microbiol..

[19] Harper M., John M., Turni C., Edmunds M., St Michael F., Adler B., Blackall P.J., Cox A.D., Boyce J.D. (2015). Development of a rapid multiplex PCR assay to genotype Pasteurella multocida strains by use of the lipopolysaccharide outer core biosynthesis locus. J. Clin. Microbiol..

[20] Ma H., Wang D., Meng F., Yuan Z., Pan C., Zhao C., Shi B., Zeng J. (2025). Prevalence and relative-risks of pasteurella in yaks of Xizang, China. Trop. Anim. Health Prod..

[21] Patiño P., Gallego C., Martínez N., Rey A., Iregui C. (2022). Intranasal instillation of Pasteurella multocida lipopolysaccharide in rabbits causes interstitial lung damage. Res. Vet. Sci..

[22] Kannaki T.R., Priyanka E., Haunshi S. (2021). Research Note: Disease tolerance/resistance and host immune response to experimental infection with Pasteurella multocida A:1 isolate in Indian native Nicobari chicken breed. Poult. Sci..

[23] Becsei Á., Solymosi N., Csabai I., Magyar D. (2021). Detection of antimicrobial resistance genes in urban air. Microbiologyopen.

[24] Porcheron G., Garénaux A., Proulx J., Sabri M., Dozois C.M. (2013). Iron, copper, zinc, and manganese transport and regulation in pathogenic Enterobacteria: Correlations between strains, site of infection and the relative importance of the different metal transport systems for virulence. Front. Cell. Infect. Microbiol..

[25] Wang X., Pu J., Liu Z., Jiang J., Shang D., Dong W. (2025). Metal ion-exchanged faujasite zeolites materials against clinically isolated multidrug-resistant bacteria. World J. Microbiol. Biotechnol..

[26] Harper M., Boyce J.D., Adler B. (2006). Pasteurella multocida pathogenesis: 125 years after Pasteur. FEMS Microbiol. Lett..

[27] Jacques M., Bélanger M., Diarra M.S., Dargis M., Malouin F. (1994). Modulation of Pasteurella multocida capsular polysaccharide during growth under iron-restricted conditions and in vivo. Microbiology.

[28] He F., Qin X., Xu N., Li P., Wu X., Duan L., Du Y., Fang R., Hardwidge P.R., Li N. (2020). Pasteurella multocida Pm0442 Affects Virulence Gene Expression and Targets TLR2 to Induce Inflammatory Responses. Front. Microbiol..

[29] Bosch M., Garrido M.E., Llagostera M., Pérez De Rozas A.M., Badiola I., Barbé J. (2002). Characterization of the Pasteurella multocida hgbA gene encoding a hemoglobin-binding protein. Infect. Immun..

[30] Peng Z., Wang X., Zhou R., Chen H., Wilson B.A., Wu B. (2019). Pasteurella multocida: Genotypes and Genomics. Microbiol. Mol. Biol. Rev..

[31] Kim E.J., Oh E.K., Lee J.K. (2015). Role of HemF and HemN in the heme biosynthesis of Vibrio vulnificus under S-adenosylmethionine-limiting conditions. Mol. Microbiol..

[32] Simpson W., Olczak T., Genco C.A. (2000). Characterization and expression of HmuR, a TonB-dependent hemoglobin receptor of Porphyromonas gingivalis. J. Bacteriol..

[33] Garrido M.E., Bosch M., Medina R., Llagostera M., Pérez de Rozas A.M., Badiola I., Barbé J. (2003). The high-affinity zinc-uptake system znuACB is under control of the iron-uptake regulator (fur) gene in the animal pathogen Pasteurella multocida. FEMS Microbiol. Lett..

[34] Tubulekas I., Hughes D. (1993). Growth and translation elongation rate are sensitive to the concentration of EF-Tu. Mol. Microbiol..

[35] Keller M.R., Soni V., Brown M., Rosch K.M., Saleh A., Rhee K., Dörr T. (2025). Sugar phosphate-mediated inhibition of peptidoglycan precursor synthesis. mBio.

[36] Kroll J.S., Loynds B., Brophy L.N., Moxon E.R. (1990). The bex locus in encapsulated Haemophilus influenzae: A chromosomal region involved in capsule polysaccharide export. Mol. Microbiol..

[37] Fang X., Yuan M., Zheng M., Guo Q., Yang Y., Yang Y., Liang X., Liu J., Fang C. (2024). Deletion of glycosyltransferase galE impairs the InlB anchoring and pathogenicity of Listeria monocytogenes. Virulence.

[38] Provost M., Harel J., Labrie J., Sirois M., Jacques M. (2003). Identification, cloning and characterization of rfaE of Actinobacillus pleuropneumoniae serotype 1, a gene involved in lipopolysaccharide inner-core biosynthesis. FEMS Microbiol. Lett..

[39] Khonsari S., Cossu A., Vu M., Roulston D., Marvasi M., Purchase D. (2025). Biosurfactant-Mediated Inhibition of Salmonella Typhimurium Biofilms on Plastics: Influence of Lipopolysaccharide Structure. Microorganisms.

[40] Hummels K.R. (2025). The regulation of lipid A biosynthesis. J. Biol. Chem..

[41] Zhang J., Yin S., Yi D., Zhang H., Li Z., Guo F., Chen C., Fang W., Wang J. (2017). The Brucella melitensis M5-90ΔmanB live vaccine candidate is safer than M5-90 and confers protection against wild-type challenge in BALB/c mice. Microb. Pathog..

[42] Che J., Liu B., Fang Q., Hu S., Wang L., Bao B. (2025). Role of msbB Gene in Physiology and Pathogenicity of Vibrio parahaemolyticus. Microorganisms.

[43] Post D.M., Ketterer M.R., Phillips N.J., Gibson B.W., Apicella M.A. (2003). The msbB mutant of Neisseria meningitidis strain NMB has a defect in lipooligosaccharide assembly and transport to the outer membrane. Infect. Immun..

[44] Cesur M.F., Siraj B., Uddin R., Durmuş S., Çakır T. (2019). Network-Based Metabolism-Centered Screening of Potential Drug Targets in Klebsiella pneumoniae at Genome Scale. Front. Cell Infect. Microbiol..

[45] Yan K., Liu T., Duan B., Liu F., Cao M., Peng W., Dai Q., Chen H., Yuan F., Bei W. (2020). The CpxAR Two-Component System Contributes to Growth, Stress Resistance, and Virulence of Actinobacillus pleuropneumoniae by Upregulating wecA Transcription. Front. Microbiol..

[46] Yan X., Zhou Y., Liu S., Gu C., Xiao W., Zhao M., Yu Z., He L. (2025). Combined transcriptome and metabolome analysis reveals the regulatory network of histidine kinase QseC in the two-component system of Glaesserella parasuis. Front. Microbiol..

[47] Zolfaghar I., Evans D.J., Fleiszig S.M. (2003). Twitching motility contributes to the role of pili in corneal infection caused by Pseudomonas aeruginosa. Infect. Immun..

[48] Burrows L.L. (2012). Pseudomonas aeruginosa twitching motility: Type IV pili in action. Annu. Rev. Microbiol..

[49] Xicohtencatl-Cortes J., Monteiro-Neto V., Ledesma M.A., Jordan D.M., Francetic O., Kaper J.B., Puente J.L., Girón J.A. (2007). Intestinal adherence associated with type IV pili of enterohemorrhagic Escherichia coli O157:H7. J. Clin. Investig..

[50] Luna Rico A., Zheng W., Petiot N., Egelman E.H., Francetic O. (2019). Functional reconstitution of the type IVa pilus assembly system from enterohaemorrhagic Escherichia coli. Mol. Microbiol..

[51] Roberge N.A., Burrows L.L. (2024). Building permits-control of type IV pilus assembly by PilB and its cofactors. J. Bacteriol..

[52] McCallum M., Tammam S., Khan A., Burrows L.L., Howell P.L. (2017). The molecular mechanism of the type IVa pilus motors. Nat. Commun..

[53] Hohl M., Banks E.J., Manley M.P., Le T.B.K., Low H.H. (2024). Bidirectional pilus processing in the Tad pilus system motor CpaF. Nat. Commun..

[54] Clock S.A., Planet P.J., Perez B.A., Figurski D.H. (2008). Outer membrane components of the Tad (tight adherence) secreton of Aggregatibacter actinomycetemcomitans. J. Bacteriol..

[55] Evans S.L., Peretiazhko I., Karnani S.Y., Marmont L.S., Wheeler J.H.R., Tseng B.S., Durham W.M., Whitney J.C., Bergeron J.R.C. (2025). The structure of the Tad pilus alignment complex reveals a periplasmic conduit for pilus extension. Nat. Commun..

[56] Sasaki H., Ishikawa H., Terayama H., Asano R., Kawamoto E., Ishibashi H., Boot R. (2016). Identification of a virulence determinant that is conserved in the Jawetz and Heyl biotypes of [Pasteurella] pneumotropica. Pathog. Dis..

